# Prognosis analysis of patients with mental disorders with COVID-19: a single-center retrospective study

**DOI:** 10.18632/aging.103371

**Published:** 2020-06-19

**Authors:** Yan Wan, Juan Wu, Lihua Ni, Qinqin Luo, Cheng Yuan, Fang Fan, Hong Liu, Changjiang Zhang, Yuandi Xiang, Qin Xie

**Affiliations:** 1 Psychosis Intensive Care Unit, Affiliated Wuhan Mental Health Center, Tongji Medical College, Huazhong University of Science and Technology, Wuhan, Hubei, China; 2 Department of Dermatology, Wuhan First Hospital, Wuhan, Hubei, China; 3 Department of Nephrology, Zhongnan Hospital of Wuhan University, Wuhan, Hubei, China; 4 Department of Traditional Chinese Medicine, Wuhan First Hospital, Wuhan, Hubei, China; 5 Department of Gynecologic Oncology, Zhongnan Hospital of Wuhan University, Wuhan, Hubei, China; 6 Department of Cardiology, Enshi Tujia and Miao Autonomous Prefecture Central Hospital, Enshi, PR China; 7 Department of Otorhinolaryngology, Wuhan First Hospital, Wuhan, Hubei, China

**Keywords:** COVID-19, mental disorder, dementia, inflammatory response

## Abstract

Our study aimed to investigate the factors affecting the prognosis of patients with mental disorders with COVID-19. All patients with mental disorders who were diagnosed with COVID-19 at the intensive care unit of Wuhan Mental Health Center during the period January 3 to March 1, 2020 were selected. The influence of the baseline characteristics, clinical symptoms, laboratory parameters and the types of mental disorders on prognosis were analyzed. According to their final prognosis, the patients were divided into the deceased group (5 patients) and the cured group (25 patients). The mortality rate of patients with dementia was significantly higher than that of patients with other mental disorders (P = 0.001). The levels of certain laboratory parameters in the serum of dementia patients were significantly increased compared with levels in nondementia patients (WBC count: 10.100±6.147 vs. 5.694±3.383, p = 0.029; neutrophil count: 8.504± 5.993 vs. 3.764 ± 2.733, P = 0.008; BUN: 8.300± 4.072 vs. 4.364 ± 1.196, P = 0.001). Our research indicated that the mortality rate of dementia patients with COVID-19 was higher than that of patients with other mental disorders. A focus on the inflammatory response of dementia patients may provide novel ideas for reducing mortality.

## INTRODUCTION

An aggressive, acute respiratory disease caused by SARS-CoV-2, a novel coronavirus of zoonotic origin, called COVID-19, has become a new public health crisis threatening the world [1]. Public health and healthcare professionals are at the frontline and work hard to control and mitigate the spread of the pandemic. With the deepening of the understanding of the disease, patients with COVID-19 in various special groups have gradually attracted attention, such as cancer patients [2, 3], end-stage kidney disease patients [4], and pregnant women [5]. Patients with mental disorders need long-term treatment and specialized care, and their health and psychological status are different from those of the general population. The double hit of mental disorder and COVID-19 in the pandemic has raised great concerns. However, little information about this special group has been reported.

It has been acknowledged that mental disorders are a diverse group of conditions that primarily impair cognition, emotion, and behavioral control. Mental disorders can occur early in life and have a high aggregate prevalence in all countries, especially in low- and middle-income countries. People living with mental disorders have limited access to or difficulties understanding public health information, which predisposes them to an increased chance of infection compared with the general population. Obviously, the double hit of mental disorder and COVID-19 in the pandemic leads to increased danger. Therefore, this study focused on the factors affecting the prognosis of patients with mental disorders (especially dementia) with COVID-19.

## RESULTS

The clinical and laboratory parameters of this study are given in Table 1. The cases of a total of 30 patients were reviewed, and patients were divided into the nonsevere symptoms group (n=19) and the severe symptoms group (n=11). There were significant differences in blood oxygen saturation (95.58±1.02% vs. 88.45±3.56%, P < 0.01) and the incidence of certain clinical symptoms (anorexia/nausea, P = 0.02, and dyspnea, P < 0.01) between the nonsevere symptoms group and the severe symptoms group. However, there was no significant difference in baseline data such as age, sex, the type of mental disorder, and heart rate (P > 0.05).

**Table 1:** Baseline characteristics of mental disorders patients with COVID-19.

**Clinical parameters**	**Nonsevere symptoms group (n=19)**	**Severe symptoms group (n=11)**	**ES**	**P**
**Age(years)**	61.47±14.74	66.73±7.30	-1.01	0.28
**Gender (n, Male/Female)**	6/13	4/7	0.07	1.00
**Maximum body temperature(°C)**	37.74±1.05	38.09±1.30	-0.82	0.42
**Heart rate (bpm)**	81.42±9.78	88.55±15.48	-1.55	0.13
**Systolic pressure (mmHg)**	128.53±16.76	122.09±11.16	1.13	0.27
**Diastolic pressure (mmHg)**	79.63±8.45	77.91±11.18	0.47	0.64
**Blood oxygen saturation (%)**	95.58±1.02	88.45±3.56	8.25	**< 0.01**
**History of basic diseases (n)**				
Hypertension	11	3	2.63	0.14
Chronic bronchitis	1	0	0.60	0.22
Atherosclerosis	4	4	0.84	0.36
**Symptom (n)**				
Fever	11	8	0.66	0.42
Cough	9	9	3.45	0.06
Muscle soreness	0	1	1.79	0.18
Expectoration	3	2	0.03	0.87
Hemoptysis	0	0	-	-
Dizzy	1	2	1.29	0.26
Headache	0	0	-	-
Diarrhea	4	3	0.15	0.70
Fatigue	8	8	2.63	0.11
Pharyngalgia	0	0	-	-
Stuffy nose/Runny nose	0	0	-	-
Anorexia/Nausea	1	6	9.46	**0.02**
Dyspnea	1	10	22.00	**< 0.01**
Lumbago	0	2	3.70	0.05

ES=effect size

We regrouped the patients according to their final prognosis and divided the patients into the deceased group (n = 5) and the cured group (n = 25). There were significant differences in some clinical symptoms (fatigue, anorexia/nausea and dyspnea) and laboratory parameters (AST and BUN) between the two groups (P < 0.05, Table 2). Surprisingly, the mortality rate of patients with dementia was significantly higher than that of patients with other mental disorders (P = 0.001, Table 2).

**Table 2:** Univariate analysis based on the prognosis of patients.

**Clinical parameters**	**Cured(n=25)**	**Deceased(n=5)**	**ES**	**P**
**Age (years)**	62.72±13.640	66.80±5.070	-0.652	0.520
**Basic mental illness (n)**				
Dementia	2	3	8.112	**0.004**
Nondementia	23	2		
**Gender (n)**			1.920	0.166
Male/Female	7/18	3/2		
**Symptom severity (n)**				
Severe cases	6	5	10.364	**0.001**
Non Severe cases	19	0		
**Symptom (n)**				
Fever	14	5	3.474	0.062
Cough	15	3	0.000	1.000
Muscle soreness	1	0	0.207	0.649
Expectoration	5	0	1.200	0.273
Dizziness	2	1	0.667	0.414
Diarrhea	6	1	0.037	0.847
Fatigue	11	5	5.250	**0.022**
Anorexia/Nausea	4	3	4.509	**0.034**
Dyspnea	6	5	10.364	**0.001**
**Blood routine**				
WBC (10^9^/L)	5.877±3.419	9.184±6.720	-1.633	0.107
Neutrophil count (10^9^/L)	3.978±2.776	7.434±6.743	-1.949	0.061
Lymphocyte count (10^9^/L)	1.393±0.680	1.180±0.481	0.666	0.511
Monocyte count (10^9^/L)	0.464±0.301	0.454±0.106	0.075	0.941
Hemoglobin (g/L)	125.640±15.196	131.200±7.050	-0.793	0.435
Platelet count (10^9^/L)	180.720±49.526	143.800±34.172	1.582	0.125
Albumin(g/L)	37.550±5.534	34.180±0.838	1.199	0.241
**Blood biochemistry**				
AST (U/L)	24.360±12.086	49.250±27.585	-3.157	**0.004**
ALT (U/L)	22.760±13.758	31.250±29.296	-0.971	0.340
TBil (umol/L)	7.396±3.517	7.525±1.611	-0.071	0.944
SCr(umol/L)	76.816±43.071	111.180±56.265	-1.552	0.132
BUN (mmol/L)	4.212±1.613	9.060±3.464	-4.984	**<0.001**
UA (umol/L)	324.720±97.053	351.800±97.513	-0.569	0.574

ES=effect size; TBil=total bilirubin; SCr=serum creatinine; BUN=blood urea nitrogen; UA=uric acid

**Figure 1:**
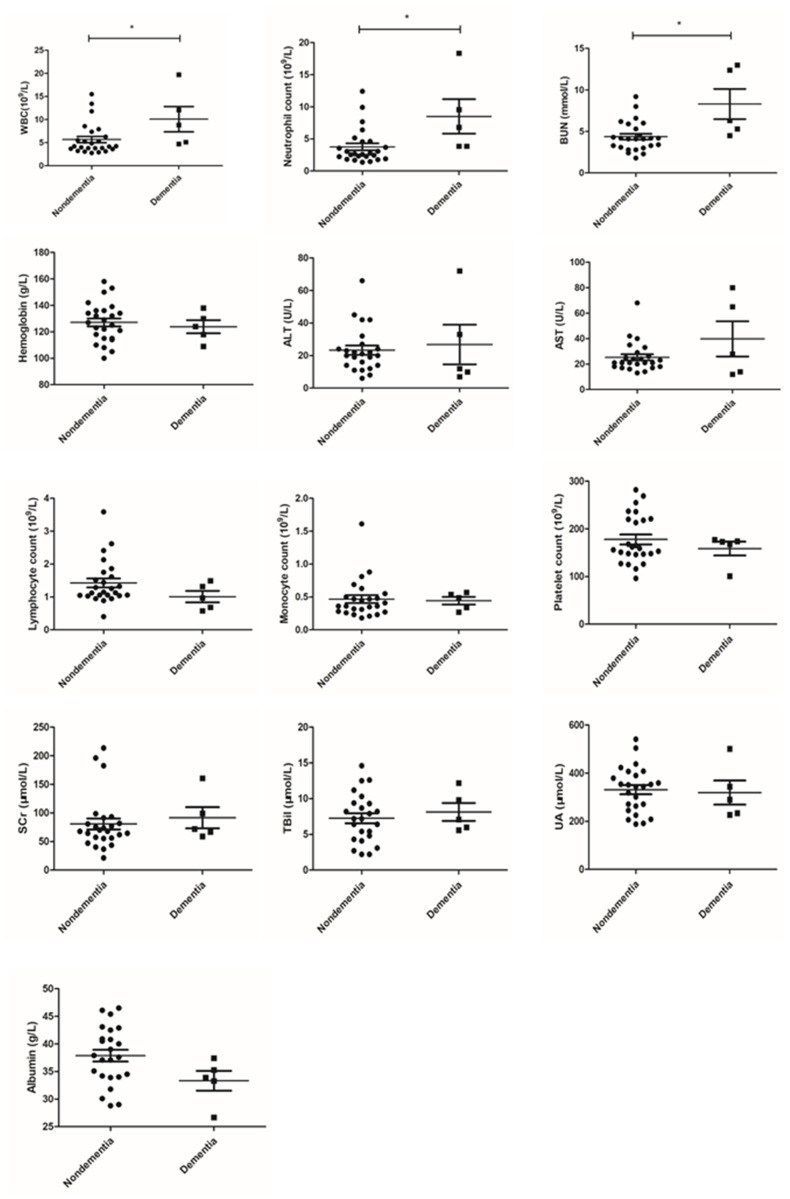
**Comparison of laboratory parameters between dementia and nondementia patients.**

To further explain this phenomenon, we compared the blood indexes of dementia patients and nondementia patients. The results showed that certain laboratory parameters in the serum of dementia patients were significantly increased (WBC count: 10.100 ± 6.147 vs. 5.694 ± 3.383, P = 0.029; neutrophil count: 8.504 ± 5.993 vs. 3.764 ± 2.733, P = 0.008; BUN: 8.300 ± 4.072 vs. 4.364 ± 1.196, P = 0.001; Figure 1).

**Figure 2:**
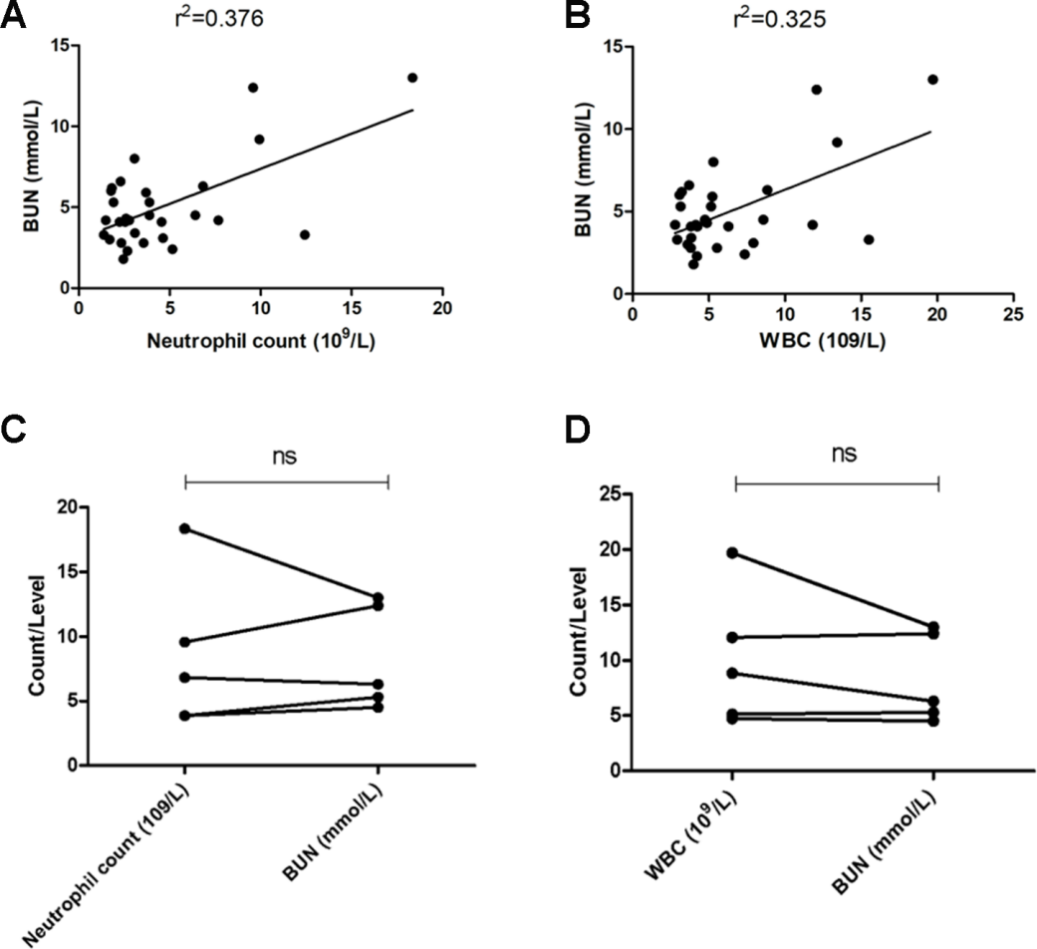
**Correlation between inflammation and renal function.** (**A**) Correlation analysis of BUN and neutrophil count in all patients with mental disorders; (**B**) correlation analysis of BUN and WBC count in all patients with mental disorders; (**C**) matched test of BUN and neutrophil count in dementia patients; (**D**) matched test of BUN and WBC count in dementia patients. ns: no significance.

Is there a correlation between the upregulation of inflammation indicators (WBC and neutrophil counts) and the impairment of renal function (BUN)? Our results suggest that WBC and neutrophil counts and BUN levels in nondementia patients were significantly positively correlated (WBC count: r^2^=0.376, P < 0.05, Figure 2A; neutrophil count: r^2^=0.325, P < 0.05, Figure 2B). However, we did not find such a significant correlation in patients with dementia (Figure 2C and 2D).

## DISCUSSION

In this study, we collected data on baseline characteristics, clinical symptoms, laboratory parameters and mental disease types in patients with mental disorders with COVID-19. The above information was used to find internal associations or differences as much as possible. Differences between patients with moderate/mild symptoms and patients with severe symptoms were mainly reflected in blood oxygen saturation, anorexia/nausea, and dyspnea. We analyzed the prognosis of patients and found that the mortality rate of dementia patients was significantly higher than that of patients with other mental disorders. In addition, the WBC count, neutrophil count and BUN level of patients with dementia were significantly higher than those of patients with other mental disorders.

Facing such a very interesting result, we needed to explore why the mortality rate of dementia patients was so high. First, dementia tends to have severe mental and behavioral symptoms. Second, due to less activity and long-term bed rest, the incidence of serious complications, such as pressure ulcers, lung infections, and cardiopulmonary insufficiency, is higher in dementia patients than in nondementia patients. Last but not least, inflammation might play an important role in the pathogenesis of dementia [6–8]. Inflammation is a characteristic of Alzheimer's disease (AD; the most important cause of dementia). AD dementia patients also have different degrees of inflammation [9]. Chronic inflammation is a common feature of various types of vascular dementia (the second most important cause of dementia) [10]. In addition to amyloid protein, inflammatory molecules, including acute inflammatory reactants and inflammatory cytokines, have been found in the cerebrospinal fluid of dementia patients [11]. Peripheral infection will also aggravate the onset and development of AD [12]. However, in another study [13] of general patients with COVID-19, patients with severe cases tended to have higher leukocyte counts and neutrophil-lymphocyte ratios. Therefore, the persistent inflammatory state of dementia patients may be the cause of the increased peripheral blood WBC and neutrophil counts in dementia patients with COVID-19.

At present, there is not enough evidence to support that the renal function damage of dementia patients with COVID-19 is worse than that of patients with other mental disorders. In our study, the inflammatory response of patients with mental disorders was associated with renal impairment, but this phenomenon was not statistically significant in patients with dementia. Therefore, the increase in BUN levels may not be unique to patients with dementia with COVID-19. It may be that these patients have severe symptoms, which lead to damage to renal function. Of course, due to the limitation of the sample size of dementia patients, the trend of the correlation analysis may be masked. Therefore, the correlation between the inflammatory response and renal function impairment in dementia patients needs to be treated with caution. At least, based on the current results and previous evidence, it is not enough to deny this conclusion.

It is worth noting that the following limitations of this study cannot be ignored. Since our research is limited to patients with mental disorders, which makes the sample size insufficient, the probability of false-positive errors seen in small-sample clinical studies is difficult to avoid. In addition, dementia may also be accompanied by other mental disorders, and the heterogeneity between patients may result in an overestimation of the statistical results. Nonetheless, our research indicated that the mortality rate of dementia patients with COVID-19 was higher than that of patients with other mental disorders. A focus on the inflammatory response of dementia may provide novel ideas for reducing mortality.

## MATERIALS AND METHODS

### Participants and materials

All patients who were diagnosed with mental disorders and with COVID-19 at the intensive care unit of Wuhan Mental Health Center during the period January 2 to March 1, 2020 were selected. The definition of the mental disorders was based on the International Classification of Diseases-10 (ICD-10). The diagnosis of COVID-19 was made according to the standards for the “Diagnosis and Treatment Scheme of New Coronavirus Infected Pneumonia” (trial version 6 [14]). Finally, a total of 30 newly diagnosed COVID-19 patients with mental disorders were considered candidates in our study. Exclusion criteria included other infectious diseases, hepatic or renal insufficiency, and malignancies. No patient received COVID-19-related antiviral or symptomatic treatment before entering this study.

### Data collection

We reviewed electronic patient records retrospectively; clinical and laboratory parameters were extracted, including sex, age, the types of mental disorders, COVID-19 nucleic acid detection, routine blood test and laboratory biochemical examination results, such as total protein (TP), alanine aminotransferase (ALT), aspartate aminotransferase (AST), creatinine (Cr), blood urea nitrogen (BUN), and uric acid (UA), and so on. These laboratory parameters were evaluated when patients first underwent laboratory tests in the hospital. The study was approved by the Ethics Committee of Wuhan Mental Health Center, and all patients provided informed consent.

### Statistical analysis

All data were analyzed by using SPSS 16.0 (SPSS Inc., Chicago, IL) and GraphPad Prism 5. Continuous variables are shown as the mean ± the standard deviation (SD), and categorical variables are shown as percentages. Before analysis, the Kolmogorov–Smirnov test was conducted to identify variable normality. Continuous variables with normal distribution were analyzed by an independent-sample t test, and non-normally distributed data were compared by a rank-sum test. P < 0.05 was considered statistically significant.

### Ethical statement

All procedures performed in studies involving human participants were in accordance with the ethical standards of the institutional and/or national research committee and with the 1964 Declaration of Helsinki and its later amendments or comparable ethical standards. Informed consent was obtained from all individual participants included in the study.
